# 6-Chloro-1-phenyl­indoline-2,3-dione: absolute structure, non-linear optical and charge-transport properties

**DOI:** 10.1107/S2056989017007630

**Published:** 2017-05-31

**Authors:** Bing Wang, Qing Lu, Qi Fang, Ting-ting Zhang, Ying-ying Jin

**Affiliations:** a State Key Laboratory of Crystal Materials, Shandong University, Jinan 250100, Shandong Province, People’s Republic of China; b School of Chemistry and Chemical Engineering, Shandong University, Jinan 250100, Shandong Province, People’s Republic of China

**Keywords:** crystal structure, absolute structure, isatin derivatives, frozen chiral conformation, SHG effect, charge-transport property

## Abstract

In the title compound, C_14_H_8_ClNO_2_, the dihedral angle between the isatin moiety (r.m.s. deviation = 0.014 Å) and the phenyl ring is 51.8 (1)°. All mol­ecules have the same ‘frozen chiral’ conformation in the non-centrosymmetric *P*2_1_2_1_2_1_ space group. A polycrystalline sample of the title compound exhibits a considerable second-order non-linear optical effect (frequency doubling of 1064 nm light to output 532 nm light). In the crystal, mol­ecules are linked by C—H⋯O hydrogen bonds, generating chains along the [100] direction. Based on a DFT calculation, [100] proves to be the most favourable direction for charge transport and the title crystal could be used as a hole-transport material because of its high hole mobility.

## Abstract 2

A polycrystalline sample of the title compound exhibits a considerable second-order non-linear optical effect (frequency doubling of 1064 nm light to output 532 nm light). In the crystal, mol­ecules are linked by C—H⋯O hydrogen bonds, generating chains along the [100] direction. Based on a DFT calculation, [100] proves to be the most favourable direction for charge transport and the title crystal could be used as a hole-transport material because of its high hole mobility.

## Chemical context   

Derivatives of isatin, also called indoline-2,3-dione, have drawn great attention for their biological and pharmacological properties such as anti­convulsant (Prakash *et al.*, 2010 ▸), anti­cancer (Abadi *et al.*, 2006 ▸) and anti-HIV (Bal *et al.*, 2005 ▸) activities. The isatin skeleton can be found in analytical reagents, pesticides and dye inter­mediates. Isatin derivatives are also versatile precursors in the synthesis of a variety of heterocyclic compounds. However, the opto-electronic properties of isatin derivatives are rarely investigated.

The crystal structures of many isatin derivatives have been reported, among the analogues of the title compound are 6-bromo-1-butyl­indoline-2,3-dione (Ji *et al.*, 2009 ▸), 1-ethyl-5-iodo­indoline-2,3-dione (Wang *et al.*, 2014 ▸), 6-chloro­indoline-2,3-dione (Golen & Manke, 2016 ▸), 1-benzyl-5-fluoro­indoline-2,3-dione (Sharmila *et al.*, 2015 ▸) and 1-phenyl­indoline-2,3-dione (Shukla & Rajeswaran, 2011 ▸). The synthesis of the title compound was reported in 2014 (Bergman & Stensland, 2014 ▸). Recently, we prepared this compound by a different method, which involves the use of O_2_ in air as oxidant. Herein, we report the crystal structure and some opto-electronic properties of this compound.
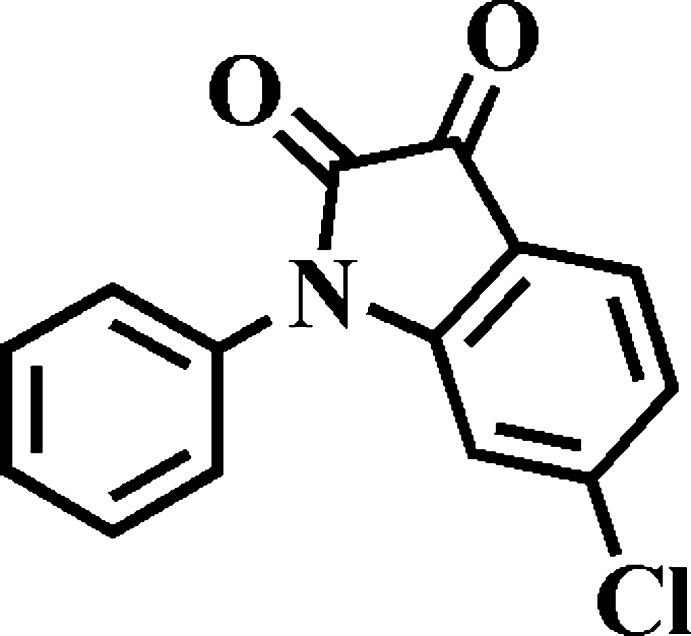


## Structural commentary   

**Figure 1:**
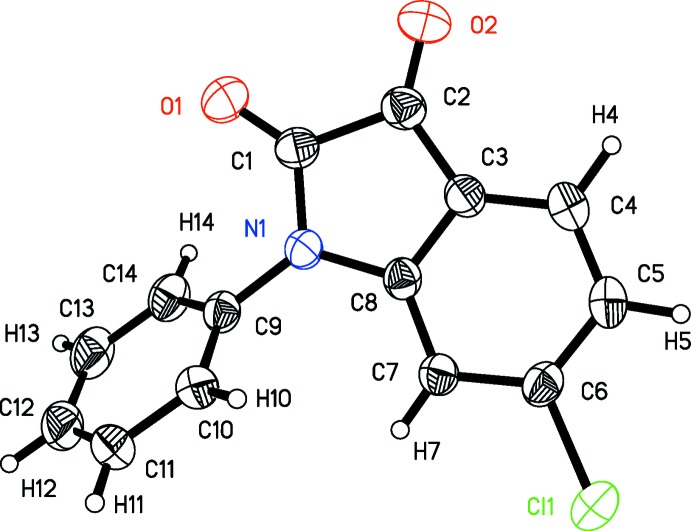
The mol­ecular structure of the title compound, with displacement ellipsoids shown at the 50% probability level.

As shown in Fig. 1 ▸, the isatin unit of the mol­ecule is essentially planar, with a mean deviation of 0.009 (2) Å and a maximum deviation of 0.0870 (8) Å (for atom O1) from the mean plane of the indoline core (C1–C8/N1). As a result of the short intra­molecular contacts (C10⋯C7, C14⋯O1) and the H7⋯H10 steric hindrance, there is a dihedral angle of 51.8 (1)° between the phenyl ring and the mean plane of the indoline core. As a comparison, the dihedral angle of the DFT/b3lyp/6-311++g(2d,p) optimized (see below) title mol­ecule is 60.0°. The sum of the angles surrounding N1 is 359.96°, suggesting that this atom is *sp* ^2^ hybridized. The C9—N1 bond length [1.4279 (14) Å] is slightly shorter than that [1.436 (2) Å] in the similar compound 1-phenyl­indoline-2,3-dione (Shukla & Rajeswaran, 2011 ▸). The C1—C2 [1.557 (2) Å] bond length is longer than a typical C*sp* ^2^—C*sp* ^2^ bond but it is notable that the geometry optimization gave a length of 1.568 Å for this bond. The C1—C2 length [1.545 (3) Å] in 1-phenyl­indoline-2,3-dione is somewhat shorter (Shukla & Rajeswaran, 2011 ▸).

As a result of the *P*2_1_2_1_2_1_ space group of the crystal, all mol­ecules have the same ‘frozen chiral’ conformation (defined as conformation I). The single conformation of these mol­ecules in this as-tested crystal is confirmed by a Flack parameter *x* = 0.03 (5) and *R*_1_ factor of 0.0317. By comparison, an inversion operation to the present structure resulted in an incorrect structure of conformation II with *x* = 0.97 (5) and *R*_1_ = 0.0336. 1-Phenyl­indoline-2,3-dione also crystallized in *P*2_1_2_1_2_1_ (Shukla & Rajeswaran, 2011 ▸) and this space group may be well suited to accommodate this class of mol­ecules.

**Figure 2:**
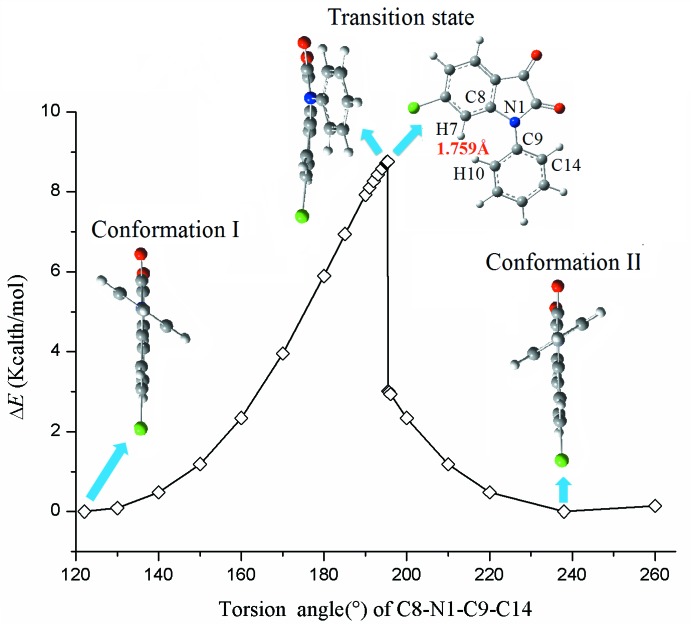
DFT/b3lyp/6–311++g(2 d,p) optimization of series of relaxed conformation with different C8—N1—C9—C14 torsion angles for the title mol­ecule.

As shown in Figs. 1 ▸ and 2 ▸, the isoenergic conformations I and II are mirror images and non-superposable one another. The calculated rotation barrier (rotating around the N1—C9 bond to transform from I to II) is 8.74 kcal mol^−1^, which is much higher than the thermal energy *k*_B_ *T* = 0.596 kcal mol^−1^ at 300 K. The main hindrance from free rotation may be the H7⋯H10 steric repulsion with a calculated distance of 1.759 Å at the transition state (see Fig. 2 ▸).

## Supra­molecular features   

**Figure 3:**
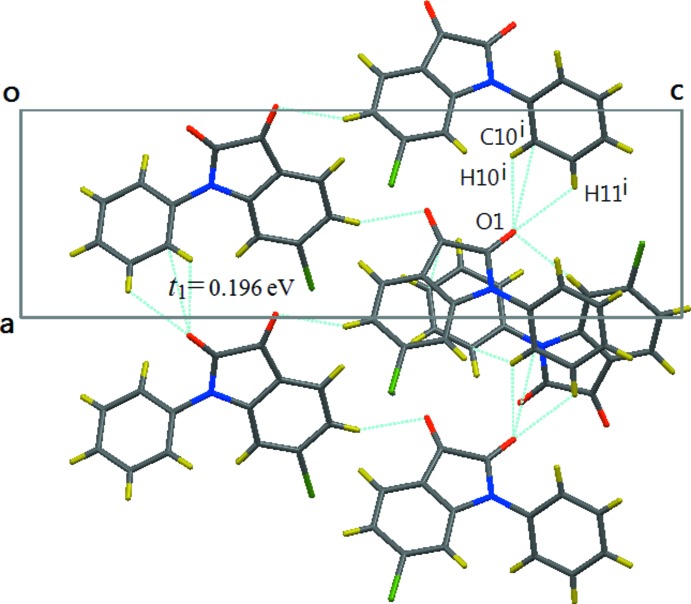
The view along the *b* axis, showing the chain linkage by the C10^i^—H10^i^⋯O1 hydrogen bond and the O1⋯H11^i^ short inter­molecular contacts along the *a*-axis direction. [Symmetry code: (i) −1 + *x*, *y*, *z*.]

As shown in Fig. 3 ▸, the inter­molecular inter­actions in the *a*-axis direction are characterized by a C10—H10⋯O1 hydrogen bond (Table 1 ▸) and an O1⋯H11(*x* − 1, *y*, *z*) [2.63 (2) Å] short contact between two side-by-side mol­ecules. The strength of the hydrogen bond can be scaled by the electronic transfer integral (*t*) between two mol­ecules and it was calculated by equation (3). The *t* value between the above two adjacent mol­ecules is maximal (*t*_1_ = 0.196 eV), indicating that a kind of side-by-side one-dimensional chain has formed along the *a*-axis direction. We believe that this *a*-directional chain plays an important role in guiding the crystal growth, for the long axis of the bar-shaped crystal was indexed to be in the [100] direction.

**Figure 4:**
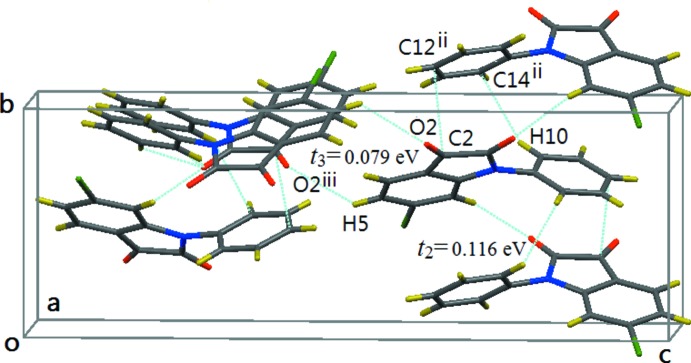
The view along the *a* axis, showing the columnar structure and short contacts of C2⋯C12^ii^ and H10⋯C14^ii^ along the *b*-axis direction, also showing the short contact of H5⋯O2^iii^ along the *c* direction. [Symmetry codes: (ii) 2 − *x*, 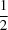 + *y*, 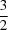 − *z*; (iii) 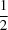 + *x*, 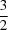 − *y*, 1 − *z*.]

By the 2_1_ [010] screw operation, mol­ecules are packed into columns along the *b*-axis direction involving C2⋯C12(2 − *x*, 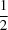 + *y*, 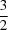 − *z*) [3.280 (2) Å] and H10⋯C14(2 − *x*, 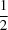 + *y*, 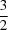 − *z*) [2.50 (3) Å] short inter­molecular contacts between two neighboring mol­ecules (see Fig. 4 ▸). The transfer integral between such two face-to-face mol­ecules is somewhat smaller (*t*_2_ = 0.116 eV) in this direction.

Along the *c*-axis direction, there is a H5⋯O2(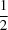 + *x*, 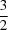 − *y*, 1 − *z*) [2.69 (2) Å] short inter­molecular contact and the *t* value between the two mol­ecules is a minimum (*t*_3_ = 0.0794 eV, see Fig. 4 ▸): thus the inter­molecular inter­actions in this direction are relatively weak.

## Calculation and opto-electronic properties   

It is well known that the necessary structural condition for second-order non-linear optical response is non-centrosymmetry, both for mol­ecules and crystals. The *P*2_1_2_1_2_1_ space group of the crystal prompted us to make a SHG (second harmonic generation) test. When the sample of crystalline powder was irradiated with infrared laser pulses (1064 nm), green light pulses (532 nm) could be observed.

Density functional theory (DFT) calculations for the electronic transfer integral *t* and the reorganization energy λ, were carried out using the *GAUSSIAN03* program (Frisch *et al.*, 2003 ▸) within the framework of b3lyp/6-311g(d).

The charge transport in organic semiconductors can be described by the hopping of an electron between a mol­ecule and a neighbouring cation (hole) or anion shown below
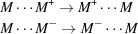



Based on the Marcus electron-transfer theory (Marcus, 1993 ▸), the mobility (μ) in a one-dimensional uniform structure, can be expressed as (Sakanoue *et al.*, 1999 ▸; Fang *et al.*, 2015 ▸)
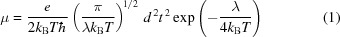
where *d* is the distance between two neighbouring mol­ecules and λ is reorganization energy. For the hole transport, λ can be expressed by (Berlin *et al.*, 2003 ▸)
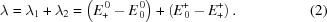
Thus, λ_1_ measures the energy difference between the stable mol­ecule and the mol­ecule with the cation geometry and λ_2_ measures the energy difference between the stable cation and the cation with the mol­ecule geometry.

The *t* in equation (1) is the electronic transfer integral, which measures the inter­molecular inter­actions between two neighbouring mol­ecules and can be calculated by (Deng & Goddard, 2004 ▸)
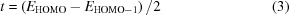
where *E*_HOMO_ and *E*_HOMO-1_ are the energy levels of the HOMO (highest occupied mol­ecular orbital) and the HOMO-1 orbital of a two-mol­ecule pair, respectively.

**Figure 5:**
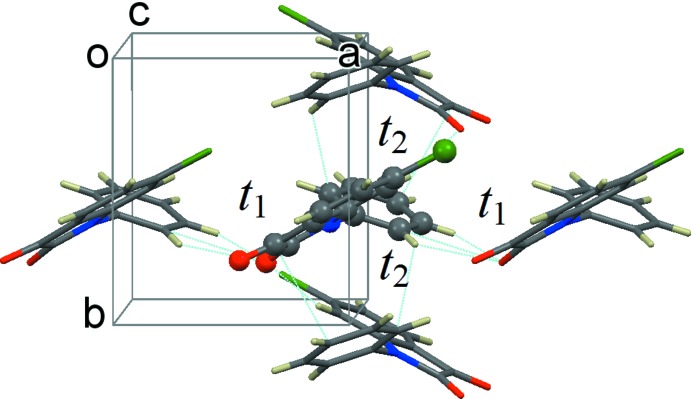
The view along the *c* axis, showing the cage-model for the DFT geometry optimization with one host mol­ecule being surrounded by four guest mol­ecules.

The mol­ecular geometry for the *t* calculation is based on this X-ray structure without optimization, while the geometries of the mol­ecule and the cation/anion have been optimized for the λ calculation. Since the mol­ecule in the crystal is different from the free mol­ecule, we adopted the cage model (Fang *et al.*, 2015 ▸) in the course of geometry optimization, in which the host (mol­ecule or cation or anion) being optimized is constrained by four guest mol­ecules with fixed X-ray structures (see Fig. 5 ▸).

As shown in Table 2 ▸, (i) the hole mobility (μ_h_) is one order of magnitude larger than the electron mobility (μ_e_), indicating that the title crystal could be used as a hole-transport material rather than an electron-transport material and (ii) both the hole mobility (μ_h_) and the electron mobility (μ_e_) in the [100] direction (the side-by-side chain direction) are an order of magnitude larger than those in the [010] direction (the face-to-face column direction).

In summary, the side-by-side hydrogen bonding in the one-dimensional chain in the [100] direction is stronger than the face-to-face π–π inter­actions in the [010] direction for this crystal, which relates to the non-linear optical and electronic transport properties of the crystal.

## Database survey   

A search in the Cambridge Structural Database (WebCSD, Version 1.1.2; last update November 2016), for indoline-2,3-dione derivatives gave 137 hits. Among them, there are nine hits for halogen 6-substituted indoline-2,3-dione derivatives and two hits which contain the substructure of the 1-phenyl­indoline-2,3-dione skeleton. There are four non-centrosymmetric structures and seven centrosymmetric structures among these eleven crystal structures.

## Synthesis and crystallization   

**Figure 6:**
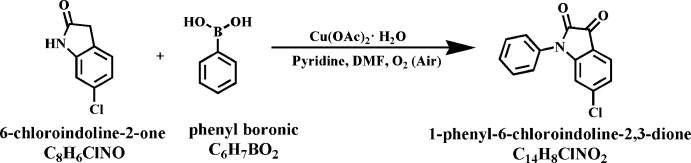
Reaction scheme.

**Figure 7:**
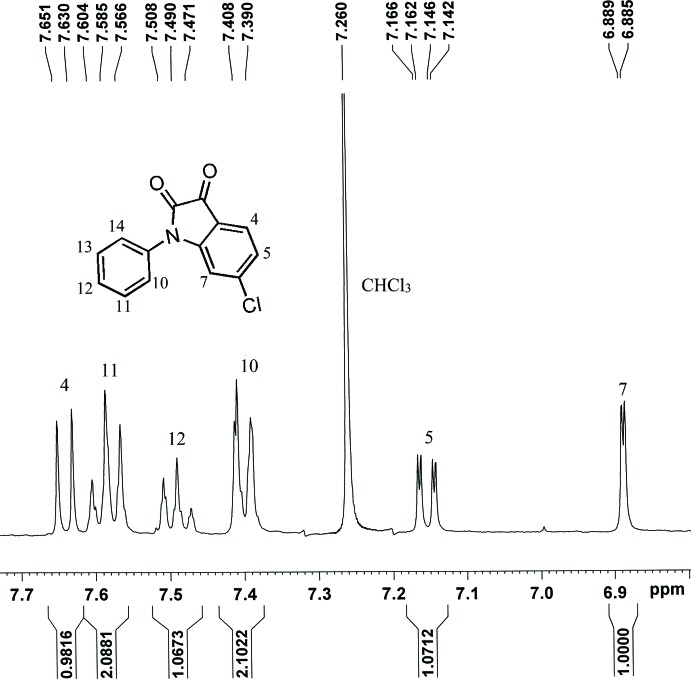
The ^1^H NMR spectra of the title compound.

We synthesized the title compound by the reaction of 6-chloro­indoline-2-one and phenyl­boronic acid (see Fig. 6 ▸). 6-Chloro­indoline-2-one (0.168 g, 1.00 mmol) was dissolved in DMF (18 ml). Then pyridine (0.05 mL), phenyl­boronic acid (0.244 g, 2.00 mmol) and Cu(OAc)_2_·H_2_O (0.197 g, 0.99 mmol) were sequentially added into the flask. The mixture was stirred for two h at room temperature in the presence of air. After filtration, the filtrate was poured into 100 ml water and extracted with di­chloro­methane. The organic phase was washed by water and dried by anhydrous Na_2_SO_4_. The crude product was purified by silica gel chromatography, eluting with a mixture of petroleum ether:ethyl acetate (30:1) to obtain an orange solid (0.096 g, yield 37%). ^1^H NMR (400 MHz, CDCl_3_) δ 7.64 (*d*, *J* = 8.4 Hz, 1H), 7.59 (*t*, *J* = 7.6 Hz, 2H), 7.49 (*t*, *J* = 7.4 Hz, 1H), 7.40 (*d*, *J* = 7.2 Hz, 2H), 7.15 (*dd*, *J* = 8.0, 1.6 Hz, 1H), 6.89 (*d*, *J* = 1.6 Hz, 1H). As shown in Fig. 7 ▸, the ^1^H NMR signals of all protons of the compound are well separated and well characterized. Orange bar-shaped crystals were obtained by slow evaporation of a solution of the title compound in mixed solvents of di­chloro­methane and *n*-hexane.

## Refinement   

Crystal data, diffraction data and structure refinement details are summarized in Table 3 ▸. All hydrogen atoms were located from the difference-electron-density maps and refined freely, resulting in C—H lengths ranging from 0.92 (2) to 1.00 (2) Å.

## Supplementary Material

Crystal structure: contains datablock(s) I. DOI: 10.1107/S2056989017007630/hb7661sup1.cif

Structure factors: contains datablock(s) I. DOI: 10.1107/S2056989017007630/hb7661Isup2.hkl

Supporting information file. DOI: 10.1107/S2056989017007630/hb7661Isup3.cml

CCDC reference: 1528555

Additional supporting information:  crystallographic information; 3D view; checkCIF report

## Figures and Tables

**Table 1:** Hydrogen-bond geometry (Å, °)

*D*—H⋯*A*	*D*—H	H⋯*A*	*D*⋯*A*	*D*—H⋯*A*
C10—H10⋯O1^i^	0.956 (17)	2.572 (18)	3.2063 (16)	124.0 (13)

Symmetry code: (i) 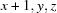.

**Table 2:** Charge-transport properties (eV, cm^2^ V^−1^ s^−1^) of the title crystal

	*t*	λ_h_ (λ_e_)	μ_h_ (μ_e_)
side-by-side [100]	0.196	0.319 (0.520)	4.67 (0.524)
face-to-face [010]	0.116	0.319 (0.520)	0.518 (0.058)

**Table 3:** Experimental details

Crystal data	
Chemical formula	C_14_H_8_ClNO_2_
*M*_r_	257.66
Crystal system, space group	Orthorhombic, *P*2_1_2_1_2_1_
Temperature (K)	294
*a*, *b*, *c* (Å)	6.8190 (3), 7.7062 (3), 21.7492 (9)
*V* (Å^3^)	1142.89 (8)
*Z*	4
Radiation type	Mo *K*α
μ (mm^−1^)	0.33
Crystal size (mm)	0.58 × 0.24 × 0.18

Data collection	
Diffractometer	Bruker APEXII CCD
Absorption correction	Multi-scan (*SADABS*; Bruker, 2005 ▸)
*T*_min_, *T*_max_	0.834, 0.943
No. of measured, independent and observed [*I* > 2σ(*I*)] reflections	21380, 3784, 3513
*R*_int_	0.021
(sin θ/λ)_max_ (Å^−1^)	0.741

Refinement	
*R*[*F* ^2^ > 2σ(*F* ^2^)], *wR*(*F* ^2^), *S*	0.032, 0.090, 1.04
No. of reflections	3784
No. of parameters	191
H-atom treatment	H atoms treated by a mixture of independent and constrained refinement
Δρ_max_, Δρ_min_ (e Å^−3^)	0.21, −0.23
Absolute structure	Flack (1983 ▸), 1583 Friedel pairs
Absolute structure parameter	0.03 (5)

Computer programs: *APEX2* and *SAINT* (Bruker, 2005 ▸), *SHELXS97*, *SHELXL97* and *SHELXTL* (Sheldrick, 2008 ▸).

## supplementary crystallographic information

### Crystal data

**Table:**

C_14_H_8_ClNO_2_	*D*_x_ = 1.497 Mg m^−^^3^
*M**_r_* = 257.66	Mo *K*α radiation, λ = 0.71073 Å
Orthorhombic, *P*2_1_2_1_2_1_	Cell parameters from 9992 reflections
*a* = 6.8190 (3) Å	θ = 2.8–31.0°
*b* = 7.7062 (3) Å	µ = 0.33 mm^−^^1^
*c* = 21.7492 (9) Å	*T* = 294 K
*V* = 1142.89 (8) Å^3^	Bar, orange
*Z* = 4	0.58 × 0.24 × 0.18 mm
*F*(000) = 528	

### Data collection

**Table:**

Bruker APEXII CCD diffractometer	3784 independent reflections
Radiation source: fine-focus sealed tube	3513 reflections with *I* > 2σ(*I*)
Graphite monochromator	*R*_int_ = 0.021
Detector resolution: 8.3 pixels mm^-1^	θ_max_ = 31.8°, θ_min_ = 1.9°
ω scans	*h* = −9→9
Absorption correction: multi-scan (SADABS; Bruker, 2005)	*k* = −10→11
*T*_min_ = 0.834, *T*_max_ = 0.943	*l* = −32→28
21380 measured reflections	

### Refinement

**Table:**

Refinement on *F*^2^	Secondary atom site location: difference Fourier map
Least-squares matrix: full	Hydrogen site location: mixed
*R*[*F*^2^ > 2σ(*F*^2^)] = 0.032	H atoms treated by a mixture of independent and constrained refinement
*wR*(*F*^2^) = 0.090	*w* = 1/[σ^2^(*F*_o_^2^) + (0.0575*P*)^2^ + 0.0865*P*] where *P* = (*F*_o_^2^ + 2*F*_c_^2^)/3
*S* = 1.04	(Δ/σ)_max_ = 0.001
3784 reflections	Δρ_max_ = 0.21 e Å^−^^3^
191 parameters	Δρ_min_ = −0.23 e Å^−^^3^
0 restraints	Absolute structure: Flack (1983), 1583 Friedel pairs
Primary atom site location: structure-invariant direct methods	Absolute structure parameter: 0.03 (5)

### Special details

**Table:**

Experimental. Scan width 0.5° ω , Crystal to detector distance 5.96 cm, exposure time 15s, 10 hours and 36 minutes for data collection, with scale. 6-run at 2theta equal -28, -28, -35,-36,-36,-38, respectively.
Geometry. All esds (except the esd in the dihedral angle between two l.s. planes) are estimated using the full covariance matrix. The cell esds are taken into account individually in the estimation of esds in distances, angles and torsion angles; correlations between esds in cell parameters are only used when they are defined by crystal symmetry. An approximate (isotropic) treatment of cell esds is used for estimating esds involving l.s. planes.
Refinement. Refinement of F^2^ against ALL reflections. The weighted R-factor wR and goodness of fit S are based on F^2^, conventional R-factors R are based on F, with F set to zero for negative F^2^. The threshold expression of F^2^ > 2sigma(F^2^) is used only for calculating R-factors(gt) etc. and is not relevant to the choice of reflections for refinement. R-factors based on F^2^ are statistically about twice as large as those based on F, and R- factors based on ALL data will be even larger.

### Fractional atomic coordinates and isotropic or equivalent isotropic displacement parameters (Å^2^)

**Table:**

	*x*	*y*	*z*	*U*_iso_*/*U*_eq_	
Cl1	1.36000 (5)	0.40110 (5)	0.561115 (17)	0.05229 (11)	
C7	1.10733 (16)	0.54301 (15)	0.64149 (5)	0.0323 (2)	
O2	0.48463 (15)	0.81725 (15)	0.61436 (5)	0.0514 (3)	
O1	0.58784 (14)	0.83661 (15)	0.74488 (5)	0.0466 (2)	
C5	1.0114 (2)	0.5438 (2)	0.53283 (6)	0.0423 (3)	
C6	1.14176 (18)	0.50392 (15)	0.57981 (5)	0.0355 (2)	
C8	0.93314 (16)	0.62745 (14)	0.65401 (5)	0.0298 (2)	
N1	0.86151 (14)	0.68187 (13)	0.71209 (4)	0.03287 (18)	
C9	0.95746 (17)	0.65986 (15)	0.76993 (5)	0.0309 (2)	
C14	0.8529 (2)	0.58710 (17)	0.81830 (6)	0.0400 (3)	
C13	0.9437 (3)	0.5724 (2)	0.87499 (6)	0.0502 (3)	
H13	0.8757	0.5247	0.9080	0.060*	
C12	1.1351 (3)	0.6281 (2)	0.88297 (6)	0.0539 (4)	
C3	0.79945 (17)	0.66972 (16)	0.60753 (5)	0.0339 (2)	
C4	0.8377 (2)	0.62747 (18)	0.54664 (6)	0.0417 (3)	
C2	0.63341 (17)	0.75880 (16)	0.63578 (6)	0.0366 (2)	
C1	0.68398 (16)	0.76703 (16)	0.70553 (6)	0.0348 (2)	
C11	1.2390 (2)	0.6983 (2)	0.83442 (6)	0.0456 (3)	
C10	1.15006 (18)	0.71514 (15)	0.77716 (5)	0.0350 (2)	
H5	1.042 (3)	0.510 (2)	0.4916 (9)	0.056 (5)*	
H4	0.739 (3)	0.659 (2)	0.5147 (9)	0.050 (5)*	
H10	1.219 (3)	0.768 (2)	0.7437 (8)	0.041 (4)*	
H11	1.369 (3)	0.735 (2)	0.8379 (9)	0.054 (5)*	
H12	1.195 (4)	0.618 (3)	0.9210 (11)	0.081 (7)*	
H14	0.724 (3)	0.548 (3)	0.8112 (9)	0.056 (5)*	
H7	1.195 (3)	0.509 (2)	0.6711 (8)	0.038 (4)*	

### Atomic displacement parameters (Å^2^)

**Table:**

	*U*^11^	*U*^22^	*U*^33^	*U*^12^	*U*^13^	*U*^23^
Cl1	0.04536 (17)	0.0669 (2)	0.04463 (17)	0.01248 (15)	0.01136 (13)	−0.00487 (15)
C7	0.0316 (5)	0.0373 (5)	0.0281 (4)	0.0001 (4)	0.0017 (4)	0.0021 (4)
O2	0.0373 (4)	0.0633 (6)	0.0536 (6)	0.0104 (4)	−0.0091 (4)	−0.0017 (5)
O1	0.0361 (4)	0.0589 (6)	0.0448 (5)	0.0057 (4)	0.0071 (4)	−0.0055 (4)
C5	0.0491 (7)	0.0514 (7)	0.0263 (5)	0.0011 (5)	0.0025 (4)	−0.0016 (5)
C6	0.0352 (5)	0.0391 (5)	0.0324 (5)	0.0011 (4)	0.0071 (4)	−0.0003 (4)
C8	0.0299 (4)	0.0330 (5)	0.0265 (4)	−0.0031 (4)	0.0022 (3)	0.0008 (4)
N1	0.0281 (4)	0.0435 (5)	0.0270 (4)	0.0015 (4)	0.0026 (3)	−0.0012 (3)
C9	0.0354 (5)	0.0315 (5)	0.0260 (4)	0.0013 (4)	0.0026 (4)	−0.0002 (4)
C14	0.0468 (7)	0.0381 (5)	0.0353 (5)	−0.0017 (5)	0.0102 (5)	0.0016 (4)
C13	0.0712 (9)	0.0490 (7)	0.0305 (6)	0.0122 (7)	0.0121 (6)	0.0080 (5)
C12	0.0714 (9)	0.0600 (8)	0.0303 (5)	0.0256 (8)	−0.0083 (6)	−0.0015 (5)
C3	0.0328 (5)	0.0384 (5)	0.0305 (5)	−0.0006 (4)	−0.0017 (4)	0.0013 (4)
C4	0.0463 (6)	0.0490 (6)	0.0299 (5)	0.0021 (5)	−0.0053 (5)	0.0005 (5)
C2	0.0313 (5)	0.0400 (5)	0.0385 (5)	−0.0019 (4)	−0.0019 (4)	0.0001 (4)
C1	0.0280 (5)	0.0393 (5)	0.0372 (5)	−0.0014 (4)	0.0017 (4)	−0.0001 (4)
C11	0.0453 (7)	0.0527 (7)	0.0388 (6)	0.0106 (6)	−0.0109 (5)	−0.0067 (6)
C10	0.0343 (5)	0.0381 (5)	0.0327 (5)	0.0017 (4)	−0.0004 (4)	−0.0005 (4)

### Geometric parameters (Å, º)

**Table:**

Cl1—C6	1.7343 (12)	C9—C10	1.3896 (16)
C7—C8	1.3815 (16)	C14—C13	1.384 (2)
C7—C6	1.3948 (15)	C14—H14	0.94 (2)
C7—H7	0.916 (17)	C13—C12	1.385 (3)
O2—C2	1.2039 (16)	C13—H13	0.9300
O1—C1	1.2040 (15)	C12—C11	1.382 (2)
C5—C4	1.3816 (19)	C12—H12	0.93 (2)
C5—C6	1.3889 (18)	C3—C4	1.3884 (17)
C5—H5	0.96 (2)	C3—C2	1.4597 (17)
C8—C3	1.3996 (15)	C4—H4	1.00 (2)
C8—N1	1.4179 (13)	C2—C1	1.5570 (17)
N1—C1	1.3844 (14)	C11—C10	1.3916 (17)
N1—C9	1.4279 (14)	C11—H11	0.93 (2)
C9—C14	1.3892 (16)	C10—H10	0.956 (17)
			
C8—C7—C6	115.85 (11)	C12—C13—H13	119.7
C8—C7—H7	123.8 (11)	C11—C12—C13	120.60 (13)
C6—C7—H7	120.3 (11)	C11—C12—H12	119.1 (16)
C4—C5—C6	119.46 (11)	C13—C12—H12	120.3 (16)
C4—C5—H5	121.2 (12)	C4—C3—C8	120.79 (11)
C6—C5—H5	119.3 (12)	C4—C3—C2	131.11 (11)
C5—C6—C7	123.52 (11)	C8—C3—C2	108.10 (10)
C5—C6—Cl1	118.54 (9)	C5—C4—C3	118.56 (11)
C7—C6—Cl1	117.94 (10)	C5—C4—H4	122.6 (11)
C7—C8—C3	121.83 (10)	C3—C4—H4	118.8 (11)
C7—C8—N1	127.67 (10)	O2—C2—C3	131.81 (12)
C3—C8—N1	110.50 (10)	O2—C2—C1	123.27 (12)
C1—N1—C8	110.46 (9)	C3—C2—C1	104.92 (10)
C1—N1—C9	123.21 (9)	O1—C1—N1	127.88 (12)
C8—N1—C9	126.29 (9)	O1—C1—C2	126.16 (11)
C14—C9—C10	121.55 (11)	N1—C1—C2	105.95 (10)
C14—C9—N1	118.68 (11)	C12—C11—C10	119.78 (14)
C10—C9—N1	119.75 (10)	C12—C11—H11	122.9 (12)
C13—C14—C9	118.54 (14)	C10—C11—H11	117.3 (12)
C13—C14—H14	122.6 (12)	C9—C10—C11	119.00 (12)
C9—C14—H14	118.8 (12)	C9—C10—H10	120.5 (10)
C14—C13—C12	120.52 (13)	C11—C10—H10	120.5 (11)
C14—C13—H13	119.7		
			
C4—C5—C6—C7	0.4 (2)	N1—C8—C3—C2	1.03 (13)
C4—C5—C6—Cl1	−179.26 (11)	C6—C5—C4—C3	0.2 (2)
C8—C7—C6—C5	−0.69 (19)	C8—C3—C4—C5	−0.45 (19)
C8—C7—C6—Cl1	178.95 (8)	C2—C3—C4—C5	178.98 (13)
C6—C7—C8—C3	0.44 (17)	C4—C3—C2—O2	1.2 (2)
C6—C7—C8—N1	179.89 (11)	C8—C3—C2—O2	−179.36 (14)
C7—C8—N1—C1	178.11 (11)	C4—C3—C2—C1	−178.96 (13)
C3—C8—N1—C1	−2.38 (13)	C8—C3—C2—C1	0.52 (13)
C7—C8—N1—C9	0.14 (18)	C8—N1—C1—O1	−176.24 (13)
C3—C8—N1—C9	179.64 (11)	C9—N1—C1—O1	1.80 (19)
C1—N1—C9—C14	52.94 (16)	C8—N1—C1—C2	2.58 (12)
C8—N1—C9—C14	−129.33 (12)	C9—N1—C1—C2	−179.37 (10)
C1—N1—C9—C10	−125.42 (12)	O2—C2—C1—O1	−3.1 (2)
C8—N1—C9—C10	52.31 (16)	C3—C2—C1—O1	176.95 (12)
C10—C9—C14—C13	0.92 (19)	O2—C2—C1—N1	178.00 (12)
N1—C9—C14—C13	−177.41 (11)	C3—C2—C1—N1	−1.90 (12)
C9—C14—C13—C12	−0.4 (2)	C13—C12—C11—C10	0.8 (2)
C14—C13—C12—C11	−0.4 (2)	C14—C9—C10—C11	−0.56 (18)
C7—C8—C3—C4	0.11 (18)	N1—C9—C10—C11	177.75 (11)
N1—C8—C3—C4	−179.42 (11)	C12—C11—C10—C9	−0.30 (19)
C7—C8—C3—C2	−179.43 (10)		

### Hydrogen-bond geometry (Å, º)

**Table:**

*D*—H···*A*	*D*—H	H···*A*	*D*···*A*	*D*—H···*A*
C10—H10···O1^i^	0.956 (17)	2.572 (18)	3.2063 (16)	124.0 (13)

Symmetry code: (i) *x*+1, *y*, *z*.
